# Regulation of gametogenesis and zoosporogenesis in *Ulva linza* (Chlorophyta): comparison with *Ulva mutabilis* and potential for laboratory culture

**DOI:** 10.3389/fpls.2015.00015

**Published:** 2015-01-26

**Authors:** Eleanor F. Vesty, Ralf W. Kessler, Thomas Wichard, Juliet C. Coates

**Affiliations:** ^1^School of Biosciences, University of BirminghamBirmingham, UK; ^2^Institute for Inorganic and Analytical Chemistry, Jena School for Microbial Communication, Friedrich Schiller University JenaJena, Germany

**Keywords:** green algae, gametogenesis, zoosporogenesis, morphogenesis, life cycle, algal–bacterial interactions, axenic culture, sporulation inhibitor

## Abstract

Green Ulvophyte macroalgae represent attractive model systems for understanding growth, development, and evolution. They are untapped resources for food, fuel, and high-value compounds, but can also form nuisance blooms. To fully analyze green seaweed morphogenesis, controlled laboratory-based culture of these organisms is required. To date, only a single Ulvophyte species, *Ulva mutabilis* Føyn, has been manipulated to complete its whole life cycle in laboratory culture and to grow continuously under axenic conditions. Such cultures are essential to address multiple key questions in *Ulva* development and in algal–bacterial interactions. Here we show that another *Ulva* species, *U. linza*, with a broad geographical distribution, has the potential to be grown in axenic culture similarly to *U. mutabilis*. *U. linza* can be reliably induced to sporulate (form gametes and zoospores) in the laboratory, by cutting the relevant thallus tissue into small pieces and removing extracellular inhibitors (sporulation and swarming inhibitors). The germ cells work as an ideal feed stock for standardized algae cultures. The requirement of *U. linza* for bacterial signals to induce its normal morphology (particularly of the rhizoids) appears to have a species-specific component. The axenic cultures of these two species pave the way for future comparative studies of algal–microbial interactions.

## INTRODUCTION

The growth and development of land plants has been extensively studied and representative model systems have been developed for molecular genetic studies in several major clades, for example, *Arabidopsis* for dicots, *Oryza/Brachypodium* for monocots, *Selaginella* for lycophytes, and *Physcomitrella* for early evolving Bryophytes (The *Arabidopsis* Genome Initiative, 2000; Goff et al., 2002; Rensing et al., 2008; Banks et al., 2011; Girin et al., 2014). This has enabled translation of the understanding of basic biological principles of plant development and evolution from models to crops (Irish and Benfey, 2004; Rensink and Buell, 2004; Coudert et al., 2010; Spannagl et al., 2011; Orman-Ligeza et al., 2014), thus improving the potential of crop plants for food and biofuel, to meet the challenges of population- and climate change. Green macroalgae (seaweeds) represent a new group of organisms with great potential for tackling the challenges of food- and fuel-security (Dibenedetto, 2012), which also cause significant environmental problems in the form of green tides and biofouling (Callow and Callow, 2006a,b; Smetacek and Zingone, 2013). However, unlike land plants, green seaweeds are under-exploited as model organisms, thus the understanding of their mechanisms of growth and development is currently severely limited.

The reason for this under-exploitation is partly due to the extreme challenges faced when growing green seaweeds under sterile laboratory conditions. It has been demonstrated for several species of green algae that the epiphytic bacterial populations with which they naturally associate are absolutely required for correct development and subsequent morphogenesis (Matsuo et al., 2003; Marshall et al., 2006; Spoerner et al., 2012). Thus, the axenic cultures that are normally required for molecular genetic/functional genomic studies in a model organism, such as transformation systems and genome/transcriptome sequencing are not straightforward to develop for green seaweeds. The greatest progress has been made with one species of Chlorophyte macroalga, *Ulva mutabilis*, [which is native to Southern Cost of Portugal and originally collected by Føyn (1958)], with (i) an established laboratory culture protocol (Stratmann et al., 1996; Wichard and Oertel, 2010), (ii) a collection of developmental mutants (Løvlie, 1968), (iii) definition of bacterial species and partially purified substances that are required for proper morphogenesis (Spoerner et al., 2012) and (iv) the isolation of factors that prevent the breakdown of leafy thallus tissue into unicellular spores/gametes (zoosporogenesis and gametogenesis, respectively, collectively “sporulation”; Nilsen and Nordby, 1975; Stratmann et al., 1996). This has paved the way for truly axenic culture of *U. mutabilis*, which will enable sequencing of this species (Spoerner et al., 2012). One general issue with seaweed culture is inducing transition between generations *via* unicellular cell types (gametes or zoospores). *U. mutabilis* produces substances that inhibit the induction of gamete- and spore-formation, ‘sporulation inhibitors’ (SI; the glycoprotein SI-1 and the low molecular weight SI-2; Stratmann et al., 1996). A third substance, the ‘swarming inhibitor’ (SWI) prevents gamete release after induction has occurred (Wichard and Oertel, 2010). Gamete induction and release can be induced in vegetative *U. mutabilis* thallus by washing and tissue fragmentation, and similarly (albeit more slowly) in *U. lactuca* (Wichard and Oertel, 2010). A similar method may also work to induce spores in *U. prolifera*, as tissue fragmentation into disks leads to spore formation (Gao et al., 2010).

*Ulva* is an economically important genus, and therefore merits a deeper understanding of its growth and developmental mechanisms at the molecular level (Wichard et al., under review). *Ulva* is a food source (Nisizawa et al., 1987; Tabarsa et al., 2012) and a potential source of biomass for fuel production (Bruhn et al., 2011). However, *Ulva* also forms nuisance algal blooms (Blomster et al., 2002; Nelson et al., 2003; Hiraoka et al., 2004; Leliaert et al., 2009; Smetacek and Zingone, 2013) and is a major biofouler (Callow and Callow, 2006a). Although certain worldwide abundant species such as the sea lettuce *U. rigida* (e.g., RFU_77) can be cultured under standardized conditions (Alsufyani et al., 2014), sporulation could not easily be synchronously induced as in tubular *Enteromorpha*-like morphotypes of the genus *Ulva* (Nilsen and Nordby, 1975; Stratmann et al., 1996).

In this paper, we sought to discover whether *Ulva* species other than *U. mutabilis* could be cultured axenically in the laboratory, and whether the signals regulating *Ulva* sporulation, morphogenesis, and development are conserved between species. We chose *U. linza*, a cosmopolitan intertidal alga found, e.g., along the coastlines of the UK and in the Yellow Sea (China; Brodie et al., 2007; Xu et al., 2013), which is a well-established model for biofouling research (Callow and Callow, 2006b) and has a partly characterized microbiome (Marshall et al., 2006). We showed that *U. linza* has the potential for standardized laboratory culture. We also highlight potential species-specific requirements for the bacterial signals required for correct morphogenesis.

## MATERIALS AND METHODS

### SAMPLING AND CULTIVATION OF *Ulva*

#### Algal strains

Haploid gametophytes from the fast-growing developmental mutant “slender” (sl) of *U. mutabilis* Føyn (mating type mt+) were used for all comparative studies with *U. linza* (Føyn, 1958; Løvlie, 1964; Fries, 1975). Vegetative and fertile sporophytic *U. linza* plants were collected in March 2013, from Llantwit Major, South Wales (51°40′N; 3°48′W). Gametogenesis and sporogenesis was induced by chopping the harvested tissue using a Zyliss^®^ Smart Clean Food Chopper.

#### Bacterial strains

*Roseobacter* sp. MS2 (Genbank EU359909) and *Cytophaga* sp. MS6 (Genbank EU359911) were cultivated in marine broth medium at 20°C on an orbital shaker. They were originally isolated from *U. mutabilis* (Spoerner et al., 2012) and stocks are stored in glycerol at -80°C.

#### Cultivation conditions

Gametophytes of *U. mutabilis* and *U. linza* were raised parthenogenetically from unmated gametes or from zooids derived from sporophytes under the standard conditions (Stratmann et al., 1996). Small germlings were grown attached to the bottom of sterile culture flasks with gas-permeable screw caps containing 100 mL *Ulva* culture medium (UCM) without antibiotics. The medium for *U. mutabilis* was routinely supplemented with the two bacterial symbionts of the algae, *Roseobacter* sp. MS2 and *Cytophaga* sp. MS6 to secure normal thallus morphogenesis. Until fertility the medium was completely exchanged weekly. Later, the medium was changed only partially (50%) to avoid premature induction of gametogenesis. The medium for experimental *U. linza* was either unsupplemented (axenic), supplemented with MS2 and MS6, or its natural bacterial flora.

*Ulva mutabilis* and experimental *U. linza* were cultivated in UCM in a 17:7 h light/dark regime at 20°C with an illumination of 60–120 μmol photons m^-2^ s^-2^ (50% GroLux, 50% day-light fluorescent tubes; OSRAM, München, Germany) and no additional aeration. Freshly collected *U. linza* thalli were washed and kept in UCM (Stratmann et al., 1996) in large tanks and boxes (>1 L), in a Sanyo MLR-351 growth cabinet with Osram Lumilux Cool White L36W/840 (36 watt, 4 ft) tubes at an illumination of 50 μmol m^-2^s^-1^.

### BIOASSAYS OF EXTRACTED SPORULATION INHIBITORS

#### Chemicals

For the extraction of the SI (SI-1, SI-2), tris (hydroxymethyl) aminomethane (Tris) was purchased from VWR (Darmstadt, Germany), HCl (37%), and EDTA (≥99.9%, p.a., ACS) were obtained from Roth (Karlsruhe, Germany). Phenol was purchased from Alfa Aeser (Karlsruhe, Germany), ethanol (99.9%, LiChroSolv) from Merck KGaA (Darmstadt, Germany) and acetone from Fluka (Sigma–Aldrich, Taufkirchen, Germany). Instant Ocean was obtained from Aquarium Systems (Sarrebourg, France). All solutions were prepared with ultrapure water purified by a reverse osmosis system (TKA, Niederelbert, Germany).

#### Preparation of crude extracts for purification of the sporulation inhibitors (SI-1, SI-2)

The established extraction protocols of the SI-1 and SI-2 by Stratmann et al. (1996) were slightly modified and applied to both *U. mutabilis* and *U. linza*.

For the extraction of the SI-1 from the growth medium, 500 mL of medium from 3 to 4 week old axenic *U. mutabilis* cultures was stirred with 50 mL phenol (saturated with 100 mmol L^-1^ Tris-HCl, 1 mmol L^-1^ EDTA, pH 7.5) in a 1 L two-neck round-bottom flask for 20 min at 20°C. After centrifugation (3800 *g*, 10 min) the phenol phase was transferred into plastic tubes. The extraction was repeated once and the phenol phases were combined. After re-extracting with 100 mL 10 mmol L^-1^ Tris-HCl (pH 8.0), the phenol phase was mixed with three volumes of acetone and subsequently incubated for 30 min at -20°C. The precipitate was collected by centrifugation (3800 *g*, 20 min, 0°C) and washed three times with pre-cooled ethanol (-20°C). After drying in the vacuum, the precipitate was suspended in 100 mmol L^-1^ Tris-HCl (pH 8.0) and stored at -20°C.

For the extraction of the SI-1 from the thallus, 2 g of minced *Ulva* sp. thalli was washed with UCM and frozen with liquid nitrogen. After grinding the thalli with a pestle, the powder was thawed and resuspended directly in 5 mL of 50 mmol L^-1^ Tris-HCl (pH 8.0). This was repeated once and subsequently thalli were mixed in 2 mL of 10 mmol L^-1^ Tris-HCl (pH 8.0) and 2 mL phenol (saturated with 100 mmol L^-1^ Tris-HCl, 1 mmol L^-1^ EDTA, pH 7.5) at 60°C for 30 min. The extraction was repeated once and the phenol phase was washed with 4 mL 10 mmol L^-1^ Tris-HCl (pH 8.0) and mixed with three volumes of acetone for ≥30 min at -20°C. After drying in a vacuum, the precipitate was suspended in 100 mmol L^-1^ Tris-HCl (pH 8.0) and stored at -20°C.

For the extraction of the SI-2 from the fluid in between the bi-layered thallus, *Ulva* thalli were washed for 15 min with ultrapure water and blotted with paper. One gram of thalli was suspended in 4 mL 10 mmol L^-1^ Tris-HCl (pH 8.0) and cut into single-layered fragments with a chopper. After centrifugation (3800 *g*, 10 min), the buffer containing SI-2 was passed through cellulose acetate filters and stored at -20°C (Stratmann et al., 1996).

#### Bioassay-guided testing of sporulation inhibitors

Fertile *Ulva* sp. thalli were harvested before noon and intensively washed with half-concentrated Instant Ocean for 15 min. According to Stratmann et al. (1996) the induction efficiency (i.e., proportion of cells differentating into gametangia) increases dramatically if sporulation is induced during the G1-cell-cycle phase, which happens before noon in synchronized cultures of *U. mutabilis*. We assumed the same was true for *U. linza* and, indeed, this was the case.

After chopping the thalli, *Ulva* fragments were washed twice in a fine sieve. The fragments (*n* = 70 ± 30) were transferred into 96 multiwell dishes (Nunc, Roskilde, Denmark) with 100 μl UCM for survey of gametogenesis. The concentration of the SIs was measured *via* dilution series of the partly purified compounds with UCM according to Stratmann et al. (1996). Due to the nature of the discrete dilution series, variance of measurement also depends on the interval of the dilution steps: a dilution series of six steps ranking from 150 units to 1 unit of the respective inhibitor was performed. One unit of the SI-1 and SI-2 is hereby defined as the concentration that inhibits completely the gametogenesis of a mature alga (i.e., fragmented thallus) completely in 1 mL of UCM for 3 days at 20°C upon induction. In parallel, samples with Tris-HCl (negative control) and with defined known amounts of SI (positive control) were tested. After 3 days of incubation the sporulation rates were determined under a Leica DMIL LED microscope equipped with a DFC 280 camera (Leica, Solms, Germany). The one-way Analysis of Variance (ANOVA) and the subsequent Tukey *post hoc* tests were performed by Minitab 16 Statistical Software (2010; State College, PA, USA: Minitab, Inc.).

### PREPARATION OF AXENIC CULTURES

For the preparation of axenic cultures of *U. linza*, gametes were purified from accompanying bacteria based on the protocol developed for *U. mutabilis* (Spoerner et al., 2012): purification was performed by phototactic movement of freshly released gametes through a narrow horizontal capillary (see also review by Wichard et al., under review) toward a light source, under strictly sterile conditions in a laminar flow hood. Sterile Pasteur pipettes with 15 cm capillaries were prepared; gametes swim to the top of the pipettes, where they are collected and applied for next run of purification through a further Pasteur pipette (**Figure 3**). In general three runs are necessary to purify the gametes form the bacteria. The final preparations of axenic gametes were routinely tested for axenicity by plating aliquots on marine broth agar (Roth, Karlsruhe, Germany) and checking for absence of bacterial colony formation.

### BIOASSAY-GUIDED TESTING OF MORPHOGENESIS INDUCING BACTERIA

Standard bioassays of the activities of the bacterial morphogenetic factors were performed in sterile 50 mL plastic tissue-culture flasks (Nuclon Surface, Nunc Int.) for both *U. linza* and *U. mutabilis* (control strain). 10 mL sterile UCM was inoculated with ~1000 freshly prepared axenic gametes. After incubation overnight at 20°C in the dark, gametes randomly attached to the bottom of the flask. Axenic gametes of *U. linza* were inoculated with a combination of *Roseobacter* sp. and *Cytophaga* sp. (cell density 10^4^ cells mL^-1^) or with the natural bacterial community. As a negative control, one flask was left without any bacteria for the complete period of the experiment. The flasks were cultured under standard light: dark conditions and analyzed under the inverted microscope during the next 21 days. The observed qualitative features were [as described by Spoerner et al. (2012)]: the presence of bubble-like cell wall protrusions; degenerating blade cells and differentiated rhizoid cells.

## RESULTS

### INDUCTION OF GAMETOGENESIS AND ZOOSPORANGENESIS

**FIGURE 1:**
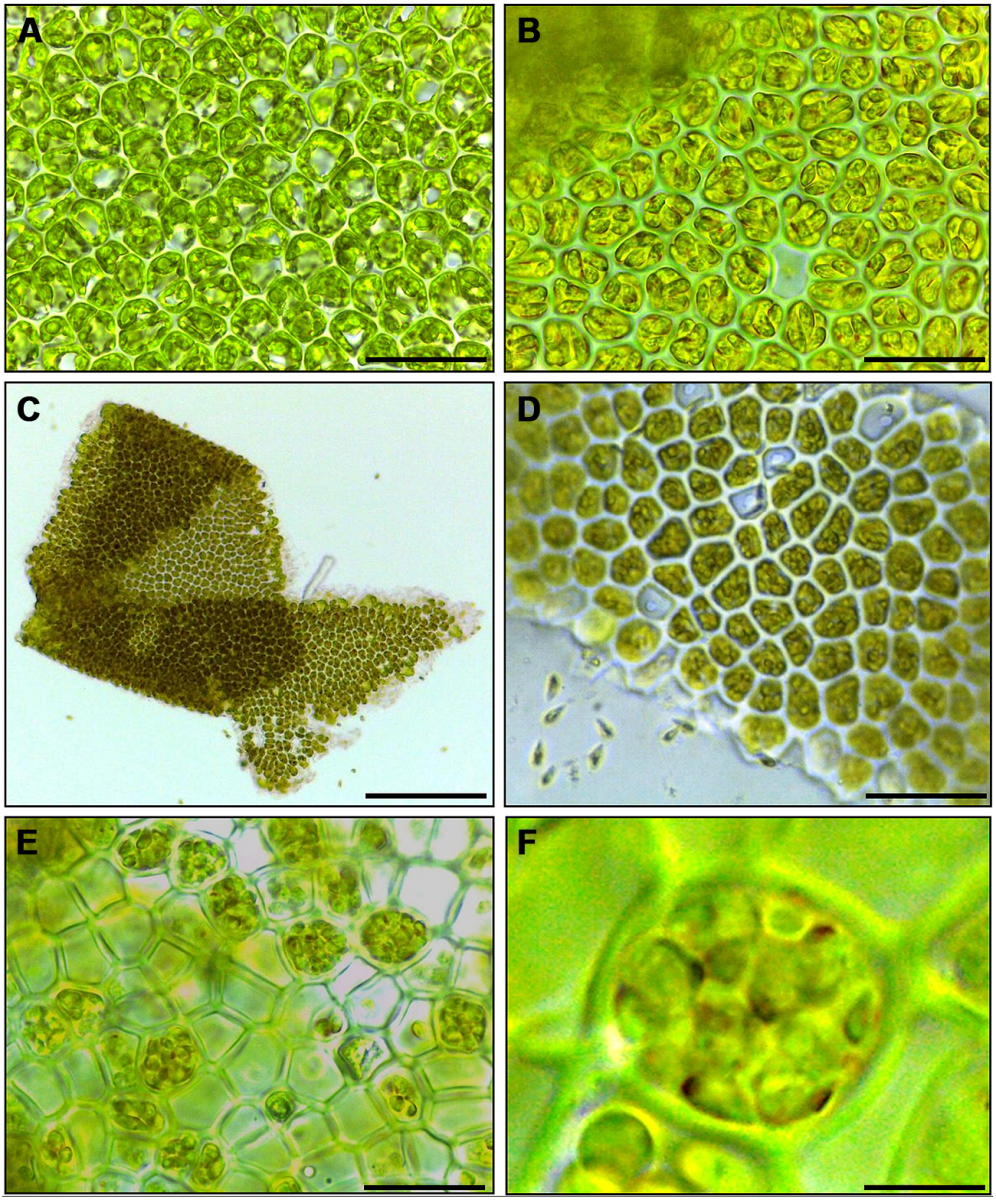
**Induction of gametogenesis and zoosporogenesis in *Ulva linza*.** Phenotypic changes of blade cells during gametogenesis and gamete release. **(A)** Blade cells 24 h after induction resemble uninduced blade cells: cells are square and often in transverse rows. **(B)** 48 h after induction, blade cells differentiate into gametangia containing finally 16 progametes, which mature during the following night into fully developed gametes ready for swarming. **(C,D)** Gametes are discharged in the morning of the third day. **(E)** Discharged sporangia and **(F)** zoospores within a sporangium are shown. Gametophytes **(A–D)** and sporophytes **(E,F)** were grown under standard conditions (Scale bars: **A,B,D** = 25 μm; **C** = 140 μm, **E** = 16 μm, **F** = 4 μm).

Cutting gametophyte blades of *U. linza* into small pieces using a chopper can induce full gamete formation and release of gametes in the morning of the third day, upon an additional medium change (**Figure 1**). On the day of induction and during the next day, the phenotype of the blade cells does not change visibly, and the orientation of the chloroplasts stays perpendicular to the light for optimal energy uptake (**Figures 1A,B**). During the second day after induction, the cells further differentiate into gametangia containing about 16 progametes, which mature during the following night into fully developed gametes ready for swarming (**Figures 1C,D**). If gametogenesis was induced in a small volume of UCM, the fully developed gametes were not released in the next morning, despite illumination, until the medium was changed again, which implies the accumulation of a SWI as reported in *U. mutabilis* (Wichard and Oertel, 2010; **Figure 1C**). In addition, applying the same protocol to sporophyte blade tissue leads to spore induction and release (**Figures 1E,F**) as observed in *U. mutabilis*. To verify the culture conditions in the laboratory, *Ulva* was grown under quasi-natural conditions, where the medium was turned over on a continuous orbital shaker: spontaneous gametogenesis was not observed until an age of 3–4 weeks or even later as previously reported by Stratmann et al. (1996).

### EXTRACTION OF SPORULATION INHIBITORS FROM *U. linza*

**FIGURE 2:**
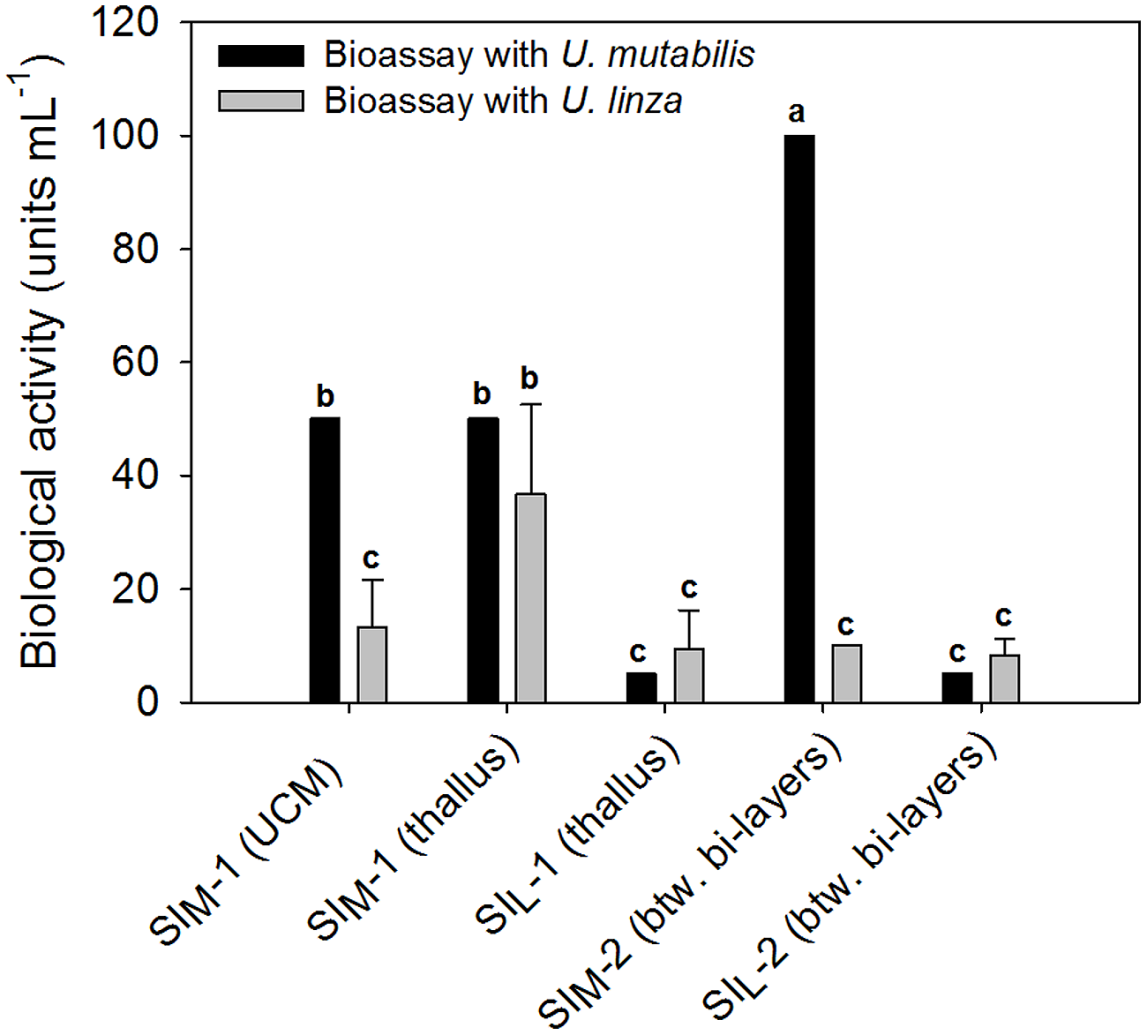
**Quantification of the sporulation inhibitors (SI), SI_M_ and SI_L_, extracted from *U. mutabilis* and *U. linza*, respectively.** SI_M_-1 was extracted from both the *Ulva* culture medium (UCM) and the thallus, whereas SI_L_-1 only from the thallus. The inhibitors were then cross-tested on both *Ulva* species (black bars = *U. mutabilis*, gray bars = *U. linza*). Activity of the inhibitors is given in units mL^-1^ (mean ± SD, *n* = 3). One unit of the SI-1 and SI-2 is defined as the concentration that inhibits the gametogenesis/zoosporogenesis of a mature alga completely in 1 mL of UCM for 3 days at 20°C upon induction. One-way ANOVA was performed to determine statistical significance. Tukey’s test was used to determine which groups differ (significance level = 5%), indicated by the letters a, b and c.

The results in Section “Induction of Gametogenesis and Zoosporangenesis” imply that sporulation in *U. linza* has similar regulation to sporulation in *U. mutabilis* and involves the removal of SI (=induction) and SWIs (=release of gametes), although they belong to different clades of *Ulva* (Guidone et al., 2013). To investigate whether gamete induction in *U. linza* requires the same or similar factors as in *U. mutabilis*, we partially purified SI from both *U. mutabilis* and freshly collected *U. linza* samples using the previously established method (Stratmann et al., 1996) and cross-tested them. We showed that *U. linza* produces SI that work interchangeably with *U. mutabilis* during gametogenesis: both types of *U. mutabilis* SI (SI_M_1 and SI_M_2) were each able to inhibit gamete production in *U. linza* and *U. mutabilis* (**Figure 2**), albeit to a lesser extent (for medium-derived SI_M_1 and between-cell-layers SI_M_2) in *U. linza*. Conversely, *U. linza* SI (SI_L_1 and SI_L_2) were each able to inhibit gamete formation in both *Ulva* species tested (**Figure 2**).

In detail, the determined biologically active concentration depends to some extent on the target species: SI_L_1 accounts for 9.4 ± 6.8 units mL^-1^ tested on *U. linza*, which was equal to 5 units mL^-1^ tested on *U. mutabilis* and thus less is less active toward *U. mutabilis*, although the difference is not statistically significant due to the high variance of the biological replicates (one-way ANOVA followed up by Tukey *post hoc* tests with an overall significance level of 5%). Moreover, SI_M_2 is significantly (about 10 times) more active when applied to *U. mutabilis* rather than to *U. linza*, but the SI_L_2 does not show any species-specific differences in its inhibitory activity (**Figure 2**).

### GENERATION OF FEEDING STOCK BY GAMETE PURIFICATION

**FIGURE 3:**
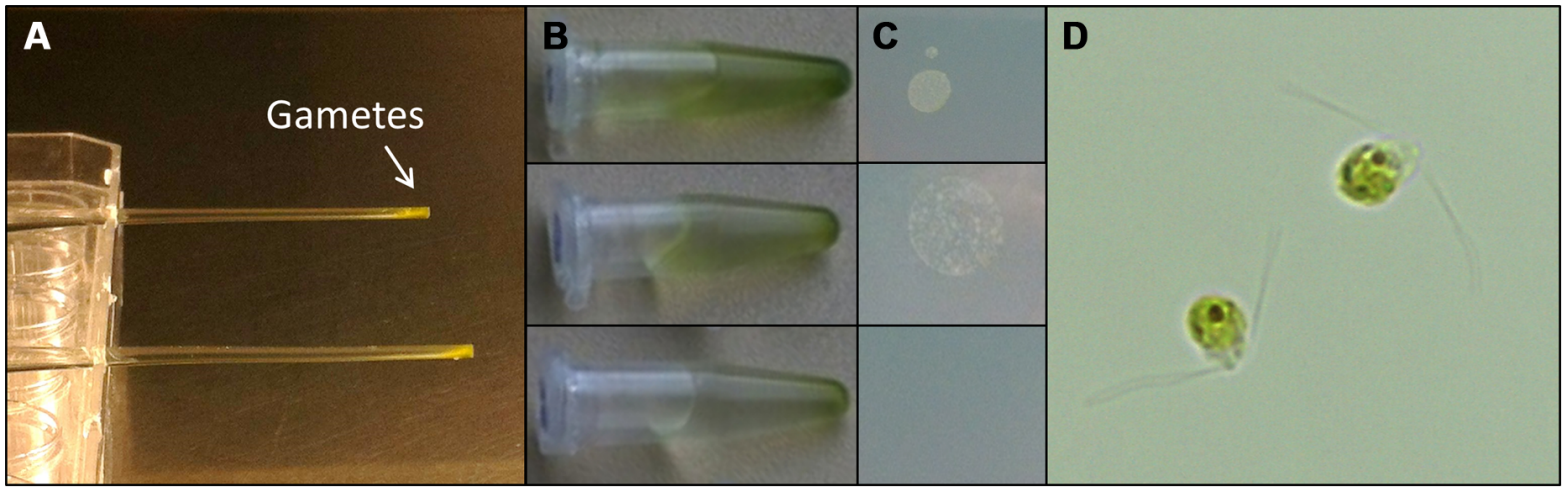
**Purification of *U. linza* gametes from accompanying bacteria.** Gametes are harvested upon medium change; capitalizing on their movement toward the light. Then collected gametes are purified in capillary pipettes several times **(A)**. The purifications can be quickly tested by placing a drop (10 μL) of the gamete solution **(B)** on marine broth agar plates **(C)**. In general three purification steps are sufficient to separate the bacteria from the gametes. Bacterial colonies forming on marine broth agar plates could be observed after two purification steps but not after the third one **(C)**. Purified bi-flagellated gametes (3 μm) are shown **(D)**.

Understanding the regulation of *U. linza* allows building up of a feedstock for further standardized cultivation, similarly to *U. mutabilis*. *U. linza* gametes from a single blade (i.e., all the same mating type) isolated upon induction of gametogenesis were able to germinate parthenogenetically to form blades. Therefore, we tried to purify *U. linza* gametes for axenic culture and to set up cultures forming thalli parthenogenetically with a controlled microbiome. As *U. mutabilis* can be put into axenic culture by purifying gametes *via* their strong and rapid phototactic response (Spoerner et al., 2012), we investigated whether *U. linza* gametes could behave (and therefore be purified) in the same way. We showed that *U. linza* gametes can be subject to purification in a very similar manner to *U. mutabilis*, over a very similar time frame (**Figure 3**). The gametes were demonstrated to be axenic by inoculation of the medium in which the purified gametes were residing onto Petri dishes: after the third purification run in Pasteur pipettes the gamete containing medium was free of bacteria (**Figure 3C**).

### BACTERIA INDUCED MORPHOGENESIS

**FIGURE 4:**
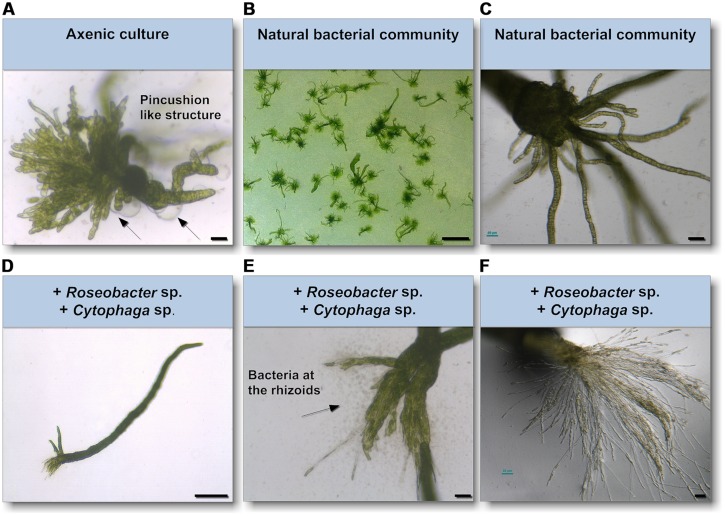
**Survey of the effects of know morphogenesis inducing bacteria on *U. linza*. (A)** Phenotype of an axenic *U. linza* gametophyte after 3 weeks cultivation with few initial thallus stages. Algae grow parthenogenetically from axenic gametes (scale bar = 25 μm). Arrows indicate the typical colorless protrusions from the exterior cell walls of axenic cultures. **(B,C)** Axenic gametes with the natural microbiome develop into a normal plant (scale bar = 1 cm in **(B)** and 50 μm in **(C)**. **(D,E)** Addition of *Roseobacter* sp. and *Cytophaga* sp. induces blade and rhizoid formation and bacterial concentrate at the rhizoids (scale bar **D** = 1 mm, **E** = 50 μm). **(F)** However, rhizoid formation seems to be incomplete in comparison of the *U. linza* grown in natural bacterial community even after 5 weeks compared to **(C)** (scale bar = 50 μm).

As epiphytic bacteria are required for correct morphology in both *U. mutabilis* and *U. linza* (Fries, 1975; Marshall et al., 2006; Spoerner et al., 2012), we tested whether *U. mutabilis* bacteria could drive the correct development of *U. linza*. Gametes were seeded in culture either axenically (purified, no bacteria), with the normal complement of *U. linza* epiphytes (i.e., gametes induced but not purified), or with the two species of bacteria known to restore morphogenesis to axenic *U. mutabilis*, namely *Cytophaga* sp. MS2 and *Roseobacter* sp. MS6 (Spoerner et al., 2012). Axenic *U. linza* formed an undifferentiated mass of cells reminiscent of axenic *U. mutabilis*, with very little cell elongation or longitudinal cell division, compared to non-axenic controls (**Figure 4**). The size of the structure formed was larger than for *U. mutabilis*. However, the callus-like morphology contained the typical colorless protrusions from the exterior cell walls as observed in axenic cultures of *U. mutabilis* (Spoerner et al., 2012), *Enteromorpha compressa* and *E. linza* (Fries, 1975).

Addition of *Roseobacter* sp. and *Cytophaga* sp. to axenic *U. linza* restored blade growth/elongation and rhizoid growth, and the bacteria clustered around the rhizoid as seen in *U. mutabilis*. However, the combination of *Roseobacter* sp. and *Cytophaga* sp. were unable to restore wild type rhizoid morphology to *U. linza.* The rhizoids formed were extremely numerous and thin, largely only one cell thick (**Figures 4E,F**). In addition, the growth of *U. linza* within this tripartite community was significantly slower than the growth of the “sl” mutant of *U. mutabilis* (3 weeks to achieve maturity; Løvlie, 1964) and the *U. linza* in a natural community.

## DISCUSSION

### GAMETOGENESIS AND SPOROGENESIS CAN BE INDUCED IN *U. linza*

We have shown that *U. linza* gametes (and zoospores) can now be reproducibly induced by cutting thallus tissue and removing extracellular inhibitors (SI-1, SI-2), as in *U. mutabilis*. This shows that there is potential for laboratory culture of a cosmopolitan *Ulva* species with worldwide distribution including in the UK, which is an established model for biofouling research and algal–bacterial interactions. A lack of gamete release when gametes are induced in a small volume of culture medium implies the existence of a SWI, as in *U. mutabilis*. The presumed release of the SWI has to be further investigated and compared with *U. mutabilis* to see whether the SWIs are exchangeable at the same concentration or whether they are even the same substance.

### *Ulva linza* PRODUCES SPORULATION INHIBITORS

We have partially purified SI (SI_L_1 and SI_L_2) from *U. linza* and compared their activity to the SI_M_ previously isolated from *U. mutabilis* (Nilsen and Nordby, 1975; Stratmann et al., 1996). *U. linza* SI_L_ works interchangeably with *U. mutabilis* during gametogenesis. *U. mutabilis* sporulation inhibitor inhibited gametogenesis in both *U. linza* and *U. mutabilis*, but to a lesser extent in *U. linza*. Conversely, *U. linza* SI inhibited gamete formation in both *Ulva* species. This demonstrates that the tested SIs are not species-specific and indicates that *U. mutabilis* and *U. linza* use similar signals to regulate induction and release of both unicellular life cycle stages. The tendency is that higher amounts of SI are necessary to inhibit the sporulation of the opposite *Ulva* species, indicating that the isolated SIs from both species are probably not identical and may differ slightly in their structure-activity relationship.

There were high variances between biological replicates with *U. linza* and this highlights the advantages of standardized culture conditions with synchronized algae. Whereas the variances were high for the bioassays with the *U. linza* due to its potentially varying age, variances of the inhibitory effects on the gametogenesis of *U. mutabilis* were so small they were not measurable within the resolution of the dilution series for three biological replicates conducted in parallel, (i.e., no SD is seen in **Figure 2**).

Compared to Stratmann et al. (1996), the extracted yield of inhibitor from the UCM (i.e., biological activity) was in general lower than previously reported. This can be explained with the lower cell densities that were used in our study. The SI_L_1 was only extracted from the thalli of vegetatively growing *U. linza* cultures and could not be detected in the UCM of *U. linza*, in contrast to *U. mutabilis* laboratory cultures. This is partly due to the fact that bacteria of the undefined microbiome of the collected *U. linza* samples have most likely digested the SI, as was also shown in natural *U. mutabilis* samples (Stratmann et al., 1996).

The observation that *U. linza* and *U. mutabilis* share similar SI and perception systems cannot be generalized within the entire *Ulva* genus, as the SI_M_1 was not effective on *U. rigida* (Stratmann et al., 1996). Because the morphology of the distromatic thalli (broad thalli with no hollow parts) of *U. rigida* is very different to the monostromatic thalli of *U. linza* and *U. mutabilis* (broad or ribbon like thalli with hollow parts), further studies need to investigate the underlying evolutionary processes and verify whether, e.g., life-cycle-regulating factors are clade-specific in the genus of *Ulva*, e.g., in the *compressa*/*pseudocurvata/mutabilis* group versus the *rigida* group (Guidone et al., 2013). Recently, it was suggested that apparently clade-specific biosynthetic pathways are used to transform polyunsaturated fatty acids into oxylipins (Alsufyani et al., 2014). Taking into account that an excess of SI-1, a cell-wall glycoprotein, is released into the environment, inhibitors might regulate the germ cell formation of closely related *Ulva* species in, e.g., tidal ponds or during green tides. This inter-species regulation might become *a fortiori* important, as waterborne breakdown products of the protein can still possess inhibitory activities (Stratmann et al., 1996; Kessler, personal communication).

In nature, the induction of sporulation might be triggered by segmentation as potentially observed by Gao et al. (2010) during green tides. Certainly sporulation events also occur even without fragmentation, whenever the SIs are either not produced or perceived in *Ulva*’s life cycle (Stratmann et al., 1996), which might explain the underlying mechanism of sporulation events reported in a recent study with a tropical *Ulva* species (Carl et al., 2014).

### GAMETE PURIFICATION AND AXENIC CULTURE OF *U. linza* AND REQUIREMENT FOR EPIPHYTES.

We have shown that *U. linza* gametes can be purified with the same methodology as developed originally for *U. mutabilis* gametes, and can germinate parthenogenetically to form new gametophyte thalli, thus paving the way for axenic culture of a second *Ulva* species. Axenic *U. linza* formed multicellular structures slightly larger than those formed by *U. mutabilis*. This could indicate species-specific differences, or could indicate a small residual (and uncultivable) bacterial load in the culture, that was not detected in the Petri dish test. As 16S PCR was not carried out, we cannot rule out this possibility.

However, when we tested the ability of the two specific bacterial species that rescue morphology in *U. mutabilis* to rescue axenic *U. linza* development, we found that recovery was slow and incomplete, particularly of the rhizoids, despite the clustering of the bacteria around the rhizoids. No filamentous basal system was formed, in contrast to Kapraun’s and Flynn’s observation with culture studies to *E. linza* (L.; Kapraun and Flynn, 1973). In summary, epiphytic bacteria are required for both growth and differentiation of *U. linza*, but *U. linza* requires different, although probably related, bacteria to *U. mutabilis* for normal morphology, particularly of rhizoid and holdfast formation. In particular, the *Cytophaga* strain releases potentially algae-specific morphogenetic substances inducing rhizoid formation in an auxin-like fashion. It supports Berglund’s (1969) studies, which found that growth of *E. linza* can in principle be stimulated by water-soluble organic substances separated from nutrients, although he did not observe changes in morphology at that time.

Marshall et al. (2006) isolated approximately 38 unique bacteria from *U. linza* and categorized them according to their morphogenetic activity within 28 days of incubation. Four categories, based on the number of tubular extensions grown from a central callus, were identified. One category holds for axenic cultures and represents a morphotype very similar to the observed axenic morphotype in this study. However, none of the other categories described the complete recovery of morphogenesis, but a combination of the isolated bacteria was not tested. Therefore, the bacteria should be re-isolated from *U. linza* according to the protocol of Marshall et al. (2006) and tested in combinations of the *Roseobacter* sp. and *Cytophaga* sp. using the newly established laboratory strains of *U. linza*.

### SUMMARY AND FUTURE WORK

We have shown that *U. linza* sporulation can be induced using the protocols previously developed for *U. mutabilis* (Stratmann et al., 1996; Wichard and Oertel, 2010; Spoerner et al., 2012), and that *U. linza* likely also produces a SWI, like *U. mutabilis*. Moreover, both species appear to use similar concepts controlling sporulation, as inhibitors purified from *U. mutabilis* and *U. linza* using identical protocols work largely interchangeably in both species. *U. linza* gametes can be purified for axenic culture and can germinate parthenogenetically, similarly to those from *U. mutabilis*. Experiments adding back *U. mutabilis* epiphytic bacteria to axenic *U. linza* gametes suggest the existence of species-specific differences in bacterial signals regulating development, particularly of rhizoids. In future, SI and SWI from *U. linza* should be further characterized, and the *U. linza*-specific bacteria and signals regulating normal development (particularly the *Cytophaga*-equivalent affecting rhizoid development) should be identified. Understanding of sporulation in more than one *Ulva* species will shed light on the formation of green tides (as seen with *U. prolifera*; Gao et al., 2010). Moreover, development of axenic culture for a second *Ulva* species potentially enables future comparative studies, particularly of the bacterial signals regulating green seaweed morphogenesis. However, our results also highlight the usefulness of a standardized model culture system using a single species for a detailed understanding of the principles of seaweed development.

## Conflict of Interest Statement

The authors declare that the research was conducted in the absence of any commercial or financial relationships that could be construed as a potential conflict of interest.
